# What are the key drivers to promote continuance intention of undergraduates in online learning? A multi-perspective framework

**DOI:** 10.3389/fpsyg.2023.1121614

**Published:** 2023-02-23

**Authors:** Jintao Zhang, Mingbo Zhang, Yanming Liu, Liqin Zhang

**Affiliations:** ^1^Faculty of Education, Jiangxi Science and Technology Normal University, Nanchang, China; ^2^School of General Education, Weifang University of Science and Technology, Weifang, China

**Keywords:** continuance intention, undergraduate, online learning, expectation confirmation theory, information system success model

## Abstract

**Introduction/Aim:**

The purpose of this study is to investigate the key predictors of online learning system continuance intention using expectation-confirmation theory and information system success model as the theoretical framework.

**Methods:**

A total of 537 respondents participated in the questionnaire to measure their self-reported responses to eight constructs (perceived usefulness, interaction, confirmation, satisfaction, continuance intention, information quality, system quality, service quality). Convenience sampling was used to obtain participants in this study. Partial least square structural equation model is used for data analysis.

**Results:**

The results showed that all the hypotheses were validated except that there was no significant positive relationship between online learning interaction and student satisfaction. Meanwhile, the variance of the continuance intention of the online learning system reached 74.0%, falling within the moderate to substantial. In addition, the multi-group analysis of perceived usefulness, satisfaction and continuance intention showed that there was no significant gender difference in the above two relationships.

**Discussion:**

Finally, this study also puts forward the theoretical and practical implications of college students’ continuance intention of online learning system.

## 1. Introduction

Online learning system can be regarded as one of the most prominent web-based educational advancements (Cheng and Yuen, 2018). According to Supriyadi et al. (2020), an online learning system, often known as “digital learning,” or “electronic learning (e-learning),” was a digital-based learning technology device such as desktop computers, laptops, tablets, and smartphones. Previous research on online learning system had mostly concentrated on issues of user satisfaction (USAT) and implementation effectiveness (Kovačević et al., 2021; Tsang et al., 2021) during COVID-19. Within the last few decades, large number of researchers argued that academics should place greater emphasis on students’ continuous use of online learning system than on their initial acceptance and subsequent success with such systems (Soria-Barreto et al., 2021; Al-Adwan et al., 2022a,b). The research on the continuous use of college students’ online learning system is divided into two parts: one is concerned with the continuous use behavior of college students’ online learning system (Al-Adwan et al., 2021, 2022a), and the other is concerned with college students’ intention to continue using their online learning system (Soria-Barreto et al., 2021; Al-Adwan et al., 2022b). According to Limayem et al. (2007), continuance intention (CI) of college students to use the online learning system is a key predictor of their continuous use behavior. Therefore, it is critical to focus on the CI of online learning systems by college students during the COVID-19 pandemic and in the period to come.

Researchers have investigated college students’ CI of online learning systems based on different theoretical frameworks, such as Expectation Confirmation Theory (ECT; Gupta et al., 2021; Wang et al., 2021), Technology Acceptance Model (TAM; Ahmad et al., 2020; Ashrafi et al., 2020; Alyoussef, 2021), The Unified Theory of Acceptance and Use of Technology (UTAUT; Liu and Pu, 2020; Wang et al., 2021), Theory of Planned Behavior (TPB; Dalvi-Esfahani et al., 2020; Baranova et al., 2022), etc. For example, based on ECT and TBP, Baranova et al. (2022) selected 301 undergraduate and postgraduate students and investigated the factors influencing CI in blended environment. The results indicated that 82% of the variance of the CI could be explained by integrating the two models. Liu and Pu (2020) took UTAUT and TAM as the theoretical framework to propose an explanatory model of learners’ CI in online learning systems. The research results showed that this model could explain 71.5% of the variance of the CI in online learning systems. The above results indicate that ECT, TAM,UTAUT,TPB, and other models are very suitable to explain the CI of college students in online learning system. In addition, a number of researchers have examined the use of online learning by students in different countries during the COVID-19 outbreak. For example, Alismaiel et al. (2022) studied the influence factors of COVID-19 on online learning and academic performance of 480 Saudi Arabian higher education students, and found that teacher-student interaction had a significant positive impact on online learning during the COVID-19 pandemic. Faura-Martínez et al. (2022) studied how 3,080 Spanish college students adapt to online learning and attend training during the COVID-19 pandemic, and the results showed that college students were not prepared for online learning, and their academic performance still declined despite spending a lot of time studying every day. Jin et al. (2021) studied 854 college students’ intention to use online learning platforms in China during the COVID-19 pandemic, and the results showed that perceived security risk, learning convenience and service quality, usability, usability, teachers’ teaching attitude, task and technology fit, and habits all significantly affected their willingness to switch from offline learning platforms to online learning platforms. Cifuentes-Faura et al. (2021) investgated the impact of COVID-19 on university students in Cambodia, Nigeria, Oman and Spain, and the results showed that COVID-19 had a negative impact on the well-being of students in these four countries, with students not receiving adequate social support and safety protection from others or teachers when they needed it. Thus, there are five major challenges facing higher education institutions during the COVID-19 pandemic: synchronous/asynchronous learning tool integration, access to technology, faculty and student online capabilities, academic dishonesty, and privacy and confidentiality (Turnbull et al., 2021).

Although previous research on CI had built on the TAM, ECT, TPB, UTAUT (Bhattacherjee, 2001; Venkatesh et al., 2003), it was found that using only constructs from these theories was inadequate to deal with the important issues of continuation intention of using technology in an online learning context. In light of this, we deemed it crucial to identify the elements of the online learning environment that motivate users to stick with it over time. According to Yan et al. (2021) ECT and information system success model (ISSM) are the two most important models for investigating the CI of college students in online learning systems. Therefore, we offered a unified model that took into account not only ECT but also the ISSM and other aspects, such as interaction (INT), that could have a distinct impact on students’ tendency to continue their online education (Lee, 2010).

The goal of this research is to better understand the factors that motivate people to keep using online learning systems. Therefore, the current study is an attempt to fill this gap by providing a comprehensive model that takes into account several antecedents of CI and putting the hypotheses to the test in the context of online education. In this study, we conduct a survey administered to undergraduate students in Chinese institution that offers courses using a hybrid model of online and traditional instruction. In addition, Partial Least Squares (PLS) is used for our data analysis needs. Therefore, the research questions of this study are as follows:

1. What factors can affect college students’ CI of online learning systems?
2. To what extent can these factors explain the total variance of college students’ CI of online learning systems?
3. Are there significant gender differences in perceived usefulness (PU), satisfaction (SAT) and CI of online learning systems for college students?

What follows is a brief overview of how the rest of this paper is structured. The literature on ECT, ISSM, and INT will be discussed in the next section. In the second section, we describe our theoretical model built on the foundation of the literature review. Methodology is addressed in the following section, and then findings and analysis of the data are presented in Section 4. We finish with a discussion of the study’s findings and their theoretical and practical implications.

## 2. Literature review

### 2.1. ECT

As a model for foreseeing and understanding SAT and continuance behavior, ECT has gained widespread acceptance in recent years (Bhattacherjee, 2001). Key predictors of SAT in ECT were user confirmation (CON) and expectations. The user’s expectations are expressed through CON, and the absence of CON indicates that those expectations were not realized. To conclude, there is a good relationship between CON and SAT (Halilovic and Cicic, 2013). Many educational research have also made use of the ECT framework to investigate into students’ CI (Wang et al., 2021).

Researchers have used ECT as a theoretical framework to study the continuance behavior of numerous types of information systems in recent years (Yang and Jiang, 2020; Wang et al., 2021). To determine if online learning technology can assist students accomplish course learning tasks during the epidemic, Yang and Jiang (2020) developed a task-technology fit (TTF) model that extends ECT. They employed a partial least squares structural equation model to test the hypotheses based on data from 854 valid responses. The findings confirmed that the theoretical framework developed for this study adequately described students’ CI in online learning context (Fu et al., 2022). In order to explain learners’ CI in a massive open online course (MOOC), Dai et al. (2020) proposed a research model that modified and extended the Expectation Confirmation Model (ECM) by including cognitive and affective variables, capturing reflections on the past and anticipations of the future, and taking into account both intrinsic and extrinsic motivations. Chinese university student data was used to test the proposed model. According to the findings, 48% of continuation intention may be explained by the proposed model. In addition, according to previous empirical findings and the theory of expectation confirmation, Lu et al. (2019) investigated the factors that contributed to user SAT and how that SAT affects their actions in MOOC. Seven factors were considered in the research: CON, usefulness, interest, flow, SAT, CI to use, and intention to recommend (ITR). For this study, a total of 300 respondents were polled. The results indicated that flow and interest were significant variables that increased MOOC SAT as measured by ECT. Recent research using ECT to study online learning is summarized in Table 1.

**Table 1:** Research topics on ECT.

Authors	Research contexts	Constructs	Fundamental theories	Key findings
Yang and Jiang (2020)	Online education platform	COM, Exception (EXP), PU, SAT, CI	ECM	CON → SAT; EXP → PU; CON → PU; PU → SAT; PU → CI; SAT → CI
Wang et al. (2021)	Online learning	CON, PU, TTF, SAT, CI	ECT + TTF	CON → PU; CON → SAT;PU → SAT; SAT → CI; PU → CI; TTF → PU
Jiang and Li (2020)	Virtual Community	Expected Confirmation (EC), SAT, PU, Perceived Service Quality (PSQ), User Experience (UE)	ECT	CON → SAT; PU → CON; PU → PSQ; PSQ → UE
Dai et al. (2020)	MOOCs	CON, usefulness (USE), SAT, CI, curiosity (CUR), attitude (ATT)	ECT	CON → ATT; CON → SAT; ATT → CI; SAT → ATT; CUR → CI
Lu et al. (2019)	MOOCs	CON, PU, SAT, CI, ITR, flow (FLO), perceived interested (PI)	ECT+ Flow theory	CON → SAT; CON → PU; CON → PI; CON → FLO; PU → SAT; PI → SAT; FLO → SAT; SAT → CI; SAT → ITR

According to the research, ECT can be utilized to describe how a student’s exposure to an online learning system impacted their real educational experience. Therefore, we adopted ECT as the framework to investigate what aspects impact learners’ SAT and CI with their online learning system.

### 2.2. ISSM

ISSM was created by DeLone and McLean (1992). Organizational impact, individual impact, use, SAT, information quality (INQ), and system quality (SYQ) are the six components of this model that evaluate the efficacy of an information system. According to the model, the constructs of system quanlity and INQ directly affected user SAT and information system usage (SU). The SAT with and use of the system affects the impact on the individual, which in turn affects the impact on the organization. To forecast information SU and user SAT, however, William and Ephraim (2003) improved and refined the original model by include another quality factor—service quality (SEQ).

A number of studies using the D&M model in an online education setting have shown varying estimates of the total variance explained by quality parameters. Recent research using D&M model to study online learning is summarized in Table 2. Empirically extending the DeLone and McLean information systems success model, Alksasbeh et al. (2019) created a model that captured the most essential quality attributes of MSN apps. It was also shown that there was a significant correlation between overall quality characteristics and student SAT and behavioral intent to use. Using the ISSM, Efiloğlu Kurt (2019) examined how students felt about a certain online learning system. The empirical findings, which were derived from the students’ self-reported perceptional evaluations of the e-learning system, confirmed that while SYQ had a significant impact on both SU and user SAT, INQ had a significant impact on user SAT but not on SU. Hsu (2021) uncovered the factors that affected the success of MOOCs for English as a Foreign Language (EFL) students (gender, age, and opened to experience). It was shown that the extent to which EFL students were willing to try new things had a substantial effect on their evaluations of the service, system, and INQ provided by MOOCs. In addition, the quality of the system was recognized as the most important factor in determining whether or not EFL students will use and be satisfied with MOOCs. Higher levels of use intention, contentment, and perceived value would result in more frequent and deeper engagement with MOOCs.

**Table 2:** Research topics on ISSM.

Authors	Research contexts	Constructs	Fundamental theories	Key findings
Efiloğlu Kurt (2019)	E-learning	SYQ, INQ, SU, USAT, system success (SS)	ISSM	USAT → SS; SU → SS; SYQ → SU; SYQ → USAT; INQ → USAT
Iqbal et al. (2022)	Digital library	INQ, SYQ, SEQ, use (USE), USAT	ISSM	SYQ → USE;INQ → SAT; SEQ → USE; SAT → USE
Xu and Du (2021)	Digital library	SYQ, INQ, SEQ, DLs’ affinity (DAF), USAT, intention to use (ITU)	ISSM + Media affinity theory	SYQ → SAT; SAT → INT
Hsu (2021)	LMOOCs	INQ, SEQ, SYQ, use intention (UINT), SAT, use of system (UOS), benefit (BEN)	ISSM	SYQ → INT; SYQ → SAT; INT → UOS; INT → SAT; UOS → BEN; SAT → BEN
Alksasbeh et al. (2019)	Mobile social networks apps	INQ, SYQ, SEQ, networking quality (NEQ), SAT, behavioral intention to use (BIU)	ISSM	INQ → SAT; SYQ → SAT; SEQ → SAT; NEQ → SAT; SAT → BIU

### 2.3. INT

It’s common knowledge that INT is crucial to effective online learning. Learner-learner INTs, learner-instructor INTs, and learner-content INTs are the three types of INTs proposed by Moore (1989), which were most commonly used to describe how people communicate in online learning or technology-based learning settings (Garrison et al., 2003; Yildiz Durak, 2018). While learner-learner INTs occur between students for the goal of sharing knowledge or ideas on course topics, learner-instructor INTs are bidirectional and help to increase or maintain students’ engagement with teaching materials. It’s useful for both mental and social appearances. Learner-content INT occurs each time a student engages with content in a way that leads to growth in that learner’s knowledge, outlook, and/or mental framework (Moore, 1989). By viewing education as a social and cognitive process, Abou-Khalil et al. (2021) argued that three types of INTs (learner-learner, learner-instructor, and learner-content INTs) were recognized as a fundamental framework to provide the minimal connections necessary for effective online learning in a crisis situation. Previous research had indicated the positive influence of these INTs on student SAT in distance education (Pham et al., 2019). Student-teacher INTs, learner-learner INTs, and learner-content INTs were all found to promote online learning performance and boost online students’ SAT with their courses by Lu et al. (2013). High-interactivity online courses are associated with higher levels of student motivation, achievement, and SAT than their less-interactive counterparts (Croxton, 2014). As a result, we hypothesize that the three forms of INTs mentioned above will have an impact on the success and SAT of students engaged in online education. According to Moore and Kearsley (1996), education, whether in-person or virtual, should focus and investigate INT. It’s a way for students to interact with their teachers, peers, and course materials to learn new things and deepen their understanding (Moore, 1989).

## 3. Hypothesis development

### 3.1. PU and CI

Davis (1989) first used the PU to describe an individual’s expectation that adopting a new system will improve his or her performance on the job. According to Bhattacherjee (2001), people do not care about the passage of time if they believe they would profit from a particular behavior. PU was found to significantly affect continuation intention, and its explanatory power was further supported by later studies (Wang et al., 2021). Furthermore, ECT-related investigations have revealed that PU influences continuation intention positively. According to the current study, when students utilize an online learning system and evaluate its utility, they are more inclined to continue using it. Therefore, Hypothesis 1 is proposed:

*H1*: PU of online learning has a significant positive relationship with the online learning CI.

### 3.2. PU and SAT

According to Zeithaml (1988), SAT is the result of a school’s success. PU is correlated strongly with SAT, according to studies of ECT (Yang and Jiang, 2020; Wang et al., 2021). In addition, The PU of online learning systems is an important predictor of students’ learning SAT (Wilson et al., 2021; Kerman et al., 2022). That is to say, the more agreeable and practical the online learning system is seen to be by the students utilizing it during the pandemic, the greater their level of SAT will be. Therefore, we put forward Hypothesis 2:

*H2*: PU of online learning has a significant positive relationship with students’ SAT.

### 3.3. PU and INT

Many research support the idea that students’ PU makes a direct or indirect contribution to their INT in online learning (Chang and Chen, 2020; Widjaja and Widjaja, 2022). Chang and Chen (2020) research also proved one of their hypotheses, namely that the PU of an e-learning system has a positive and significant effect on INT. Sher (2009) and Ramadiani (2019) discovered a positive and statistically significant association between learner-instructor INTs and the PU of e-learning system. In addition, Ramadiani (2019) found that learner-content and learner-instructor INTs have a substantial impact on the PU of e-learning system. In this study, once users find that the online learning system can effectively improve learning efficiency or performance, it can effectively improve the frequency and quality of INT. Therefore, we put forward Hypothesis 3:

*H3*: PU of online learning has a significant positive relationship with the INT.

### 3.4. CON and PU

Cognitive dissonance theory was used by Bhattacherjee (2001) to back up the claim that the degree of user CON has a positive effect on PU; a first-time user of an information system cannot confirm whether using the system allows for improved performance because they lack relevant experience. So the user would have low PU to the new system and it is easy to confirm. The user begins operations, and the system’s ability to provide advantages is confirmed in increments as time progresses. Consequently, people gradually adapt their initial thinking to post-anticipation (PU). In subsequent investigations, the effect of CON on PU has also been validated (Bhattacherjee, 2001). In the context of the current study, the degree of CON (the comparison between expectation and experience) generated by students using online learning during the pandemic will modify the previous expectation and change it to post expectation (PU). Thus, post expectation (PU) increases with the degree of CON (Wu and Wang, 2020). Therefore, Hypothesis 4 is proposed:

*H4*: CON of online learning has a significant positive relationship with the PU.

### 3.5. CON and SAT

Expectation and CON play a role in shaping levels of SAT. The extent of CON is derived from the gap between user expectations and actual use, whereas user SAT is derived from the level of CON (Bhattacherjee, 2001). In accordance with the ECT, user CON is a crucial requirement for SAT (Lee, 2010). CON is the evaluation of whether or not users have met their expectations in the context of the usage of information systems, and it is relevant to SAT (Halilovic and Cicic, 2013). In ECT-related literature, the effect of CON on SAT has been validated (Wang et al., 2021). The greater the degree of CON (comparison between anticipation and post-experience) of students utilizing an online learning system, the greater their feelings following the experience. Thus, the greater the CON, the more likely students are to have high SAT. Therefore, Hypothesis 5 is proposed:

*H5*: CON of online learning has a significant positive relationship with SAT.

### 3.6. INQ and SAT

INQ, which has been extensively studied in previous research, is defined as users’ perceptions of the quality of information (such as information obtained from a system, accuracy of the information, relevance of the information, timeliness, and completeness of the information) presented on a website (McKinney et al., 2002). For instance, research by Klobas and McGill (2010) and Eom et al. (2012) indicated a strong correlation between INQ and learning management system use and SAT. In addition, Nuryanti et al. (2021) indicated that INQ is a crucial predictors of learning management SU and SAT. Therefore, we may assume that improved quality of information in the online learning system will positively lead to an increase in PU, perceived SAT, and SU. Thus, we hypothesize that:

*H6*: INQ of online learning has a positive relationship with students’ SAT.

### 3.7. SYQ and SAT

SYQ is the degree of usability and task completion (Isaac et al., 2019). An online learning system’s SYQ is crucial for a positive UE (Ahn et al., 2004). In addition, it is noted as having an effect on performance attributes, function, and accessibility (McKinney et al., 2002). Urbach (2010) further demonstrated the significance of e-learning systems’ navigability, accessibility, structure, visual logic, and stability in ensuring a positive user learning experience. In addition, SYQ has a direct and significant impact on a person’s performance proposed by DeLone and McLean (2002). Therefore, the current research hypothesizes that:

*H7*: SYQ of online learning has a positive relationship with the students’ SAT.

### 3.8. INT and SAT

Numerous research support the notion that INT contributes either directly or indirectly to student SAT in a online learning context (Niu et al., 2022b). In addition to the variables of motivation, course structure, and teacher facilitation/knowledge, Baber (2020) shown that INT is the most influential factor in determining students’ perceived learning outcomes, which in turn impacts student SAT. Chen et al. (2020) were also successful in proving one of their hypotheses, namely that INT quality of an e-learning system has a positive and statistically significant impact on student SAT. In the meantime, Ares Albirru (2021) characterized the interactive learning environment in terms of communication and exploration of activities, and discovered that the INT aspect influences perceived SAT. From several previous studies already carried out, a hypothesis can be developed that INT will influence e-learning perceived SAT and PU that can be detailed as follows:

*H8*: INT of online learning has a significant positive influence on SAT.

### 3.9. SEQ and SAT

The SEQ of e-learning systems requires the responsiveness, empathy, trust, and safety of the supporting personnel. According to previous research, SEQ is vital to SAT and use (Tan et al., 2021), and in the context of online learning, SEQ has a significantly positive effect on online learning SU and students’ SAT (Pham et al., 2019). Teddy and Martha (2018) used structural equation modeling (SEM) to examine the link between SEQ and student SAT using a survey of 1,000 students from 13 universities and colleges in Riau Province. The outcome revealed a substantial positive relationship between SEQ and student SAT. Our argument is that the SEQ has an effect on both individual SAT and performance. Therefore, the current research hypothesizes that:

*H9*: SEQ of online learning has a significant positive influence on the students’ SAT.

### 3.10. SAT and CI

According to the literature on ECT, user SAT is a primary factor influencing their intention to reuse the online learning system (Oliver, 1980; Oliver and Bearden, 1985). In the discussion of research on CI and information systems, Bhattacherjee (2001) claimed that CI is mostly driven by the level of SAT given by actual use. Relevant research had demonstrated that SAT had a substantial influence on the CI (Bhattacherjee, 2001; Cheng, 2019; Lu et al., 2019). In conclusion, ECT provides adequate predictive power for the relationship between SAT and intention to continue (Wu and Wang, 2020). In the context of the current study, user likelihood to change the system decreases as SAT with the platform increases, and online learning CI increases with SAT. Therefore, Hypothesis 10 is proposed:

*H10*: SAT of online learning has a significant positive relationship with CI.

**Figure 1:**
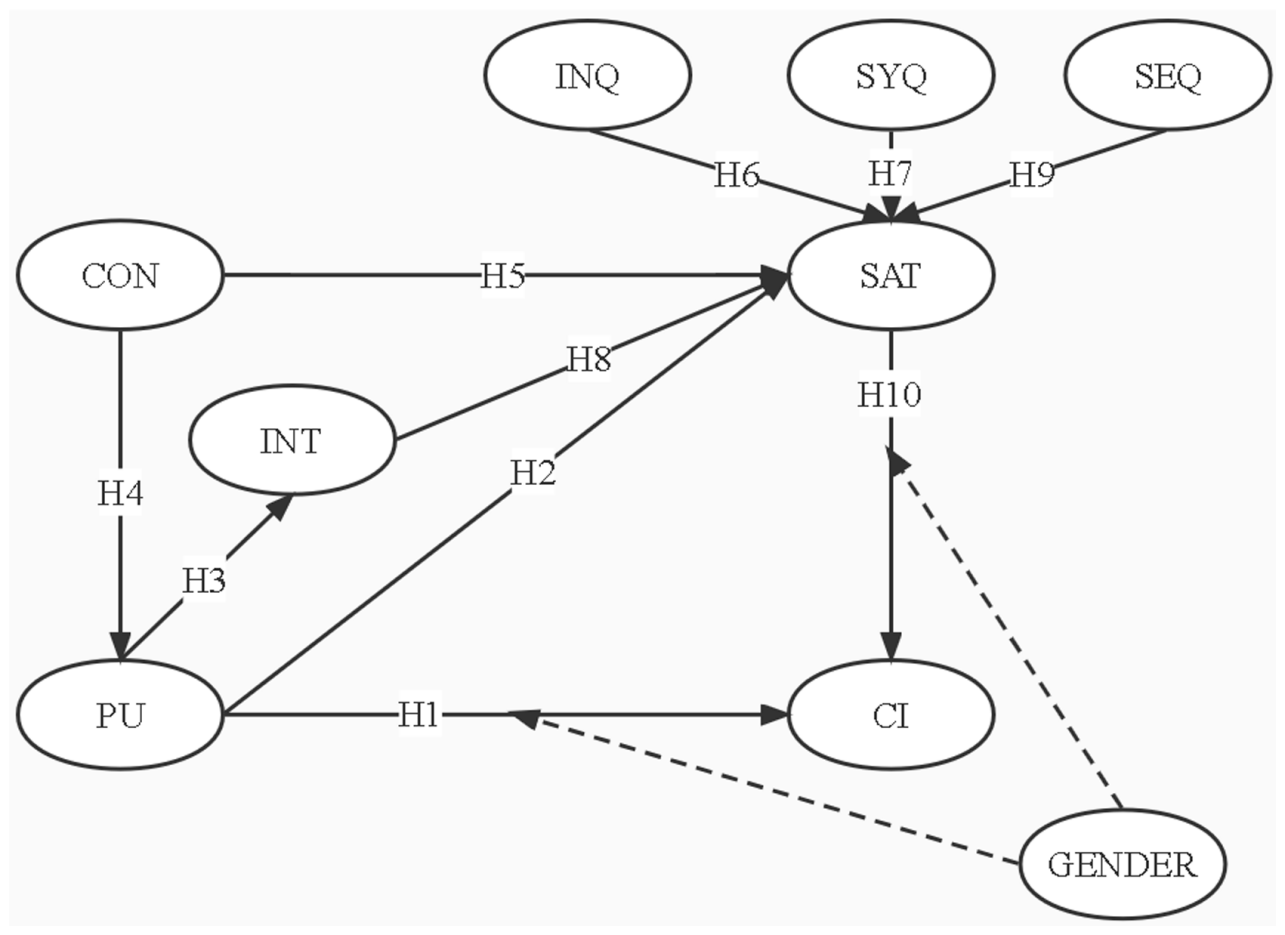
Research model. PU, perceived usefulness; INT, interaction; CON, confirmation; SAT, satisfaction; CI, continuance intention; INQ, information quality; SYQ, system quality; SEQ, service quality.

Based on the above hypothesis, the hypothesized model of this study is proposed (Figure 1).

## 4. Methodology and materials

### 4.1. Participants

This study collected a sample of three colleges (Jiangxi Science and Technology Normal University, Jiangxi University of Applied Science, and Nanchang University) in China that used Super Star, the target online learning system. Students who had utilized Super Star for at least one semester were the units of analysis in this study. In this study, we used an online survey questionnaire to gather information. Over all, 562 students participated in the survey. In total, 537 respondents were applied to validate and evaluate the research model after incomplete and invalid responses (*N* = 25) were eliminated. Adequate sampling, as described by Gravetter and Forzano (2018), increases the likelihood that participants will complete the questionnaire depending on their availability and motivation to do so. Convenience sampling was used for this study since it is one of the quickest and easiest survey methods to implement and manage. Chi-square tests for gender (male = 45.23%, female = 52.54%; *p* = 0.500) were used to assess the sample’s representativeness. No statistically significant discrepancy between the sample and population distribution was found, which indicated that the sample waw highly representative. Data were collected between May 2022 and August 2022.

The valid sample included 247 male and 290 female, 117 were freshmen, 90 were sophomores, 180 were juniors, and 150 were seniors. Among them, 67 students use the online learning system for <1 h a day, 157 students use the online learning system for 1–2 h a day, 201 students use the online learning system for 2–3 h a day, 112 students use the online learning system for 3–4 h a day.

### 4.2. Measurements

As was previously noted, the information was gathered through a web-based survey questionnaire split into two primary parts. In the first section, we asked for demographic information about each respondent. In the second part, eight constructs from the theoretical framework were evaluated. More specifically, there were 32 indicators in Part 2. The 7-point Likert scale ranges from 1 (completely disagree) to 7 (completely agree), and all questions were adopted directly from relevant literature (Appendix A).

Two methods were used to check the reliability and validity of the questionnaire before the actual data gathering. The first step involved a panel of four academic experts with extensive experience in online education evaluating the measurement instruments. Among the four assessors, there was a 90.5% level of consensus. Furthermore, the panel’s suggestions to further strengthen the study’s reliability and validity were taken into account. Secondly, a pilot research involving 60 students was conducted to assess the validity of the eight components. The results show that all constructs are reliable because their respective Cronbach’s alpha values are >0.7 (Hair et al., 2019).

Four items created by Mohammadi (2015) were adopted to assess students’ views on PU of online learning system (2015). Pursuant to the aims of the present investigation, the term “Moodle” was substituted with “online learning system” in the original scale (e.g., “Using online learning enables me to accomplish my tasks more quickly”). Three items created by Bhattacherjee (2001) were used to measure the students’ CON of online learning system. The initial scale was modified by switching “OBD” for “online learningn system” (e.g., “My experience with using online learning system was better than what I expected.”). Chung and Chen (2020)‘s six-item scale was employed to gauge participants’ INT in online learning. It mainly includes three types of INT, namely student-teacher INT (e.g., “The instructor is supportive when a student had difficulties or questions”), student–student INT (e.g., “The course foster student-to-student INT for supporting productive learning”), and student-content INT (e.g., “The course content provides mutual INT to facilitate student learning”). Three items created by Gefen et al. (2003) were adopted as a means of gauging online student SAT with their educational experiences. The original scale was modified by substituting “online learning” for “Travelocity.com” for the purposes of this research. We used a scale created by Cheng et al. (2019) to assess students’ commitment to continuing their online education. The scale developed by Cheng (2019) was what we used to determine whether or not students intended to continue their online education. The SYQ can be evaluated by how simple it is to use the system. The value and trustworthiness of the data provide metrics by which the INQ construct may be evaluated. When students have issues with the online learning system and the responsible staff responds to them quickly, with the appropriate level of expertise, and within the expected time frame, the SEQ becomes better. Urbach (2010) developed and field-tested scales to assess constructs including information, system, and SEQ.

### 4.3. Statistical analyses

This study employed a two-stage procedure for performing partial least squares (PLS) analysis. Reliability and validity analyses were performed in the first stage, while the structural model’s path coefficients and explanatory power were derived and validated in the second stage. The aforementioned two stages were conducted to ensure the constructs’ validity and reliability, as well as to check the inter-construct relationships (Anderson and Gerbing, 1988). PLS was considered because it is capable of dealing with both the model constructs and the measurement items at the same time, making it ideal for examining the causal relationships between constructs (Petter et al., 2007). Also, PLS is well-suited for dealing with the association between variables in abnormal data distribution because of its flexible criteria for the normality and random of the variables. Additionally, it is useful for evaluating complicated predictive model (Chin, 1998). This study investigated the causal relationship between PU, CON, INT, SAT, CI, INQ, SYQ, and SEQ; meanwhile, each construct contained a number of measurement items. Consequently, PLS was preferable to other SEM analysis methods for this study in order to examine the causal relationship between variables, decrease measurement errors, and prevent collinearity. In addition, according to Majchrzak et al. (2005), the minimum number of samples should be 5–10 times the number of model paths. According to the proposed criteria, the 537 samples and 6 maximal paths in this investigation are adequate for PLS analysis. The SmartPLS (Version 3.2.7) created by Ringle et al. (2015) was used in this research.

## 5. Results

Prior to actually progressing on to the measurement model, the common method variance (CMV) was assessed. Harman’s one factor test is conducted to determine the presence of CMV (Hair et al., 2019). Consequently, exploratory factor analysis (EFA) was performed. As shown by the outcome, none of these variables explained more than 50% of the variation in the measurement items. With a variance explained of 30.2%, the construct of CON exhibited the greatest variance. Consequently, it was clear that CMV was not present.

### 5.1. Outer model

At this section, the constructs and the accompanying measuring items were evaluated for validity and reliability. In accordance with Hair et al. (2019), several tests were carried out, including internal consistency reliability (Cronbach’s alpha), discriminant validity, and convergent validity. Convergent validity can be established if the average variance extracted (AVE) is >0.5 (Fornell and Larcker, 1981) and the items loadings on the intended theoretical constructs are in excess of 0.700 (Hair et al., 2021). Cronbach’s alpha and other composite reliability estimates of constructs must be >0.700 (Hair et al., 2019). Table 3 shows all items with loadings larger than the recommended value of 0.708; AVE of each construct is more than 0.5 and Cronbach’s alpha and composite reliability values >0.7. These results demonstrated that the dataset possesses good validity and reliability.

**Table 3:** Construct reliability and validity.

Construct	Item	Loading	Cronbach’s *α*	CR	AVE
PU	PU1	0.872	0.824	0.914	0.727
	PU2	0.822			
	PU3	0.834			
	PU4	0.883			
CON	CON1	0.824	0.816	0.899	0.749
	CON2	0.838			
	CON3	0.931			
INT	INT1	0.815	0.851	0.936	0.712
	INT2	0.878			
	INT3	0.815			
	INT4	0.831			
	INT5	0.811			
	INT6	0.908			
SAT	SAT1	0.845	0.847	0.882	0.714
	SAT2	0.852			
	SAT3	0.839			
CI	CI1	0.834	0.807	0.915	0.73
	CI2	0.837			
	CI3	0.845			
	CI4	0.900			
SEQ	SEQ1	0.809	0.844	0.895	0.681
	SEQ2	0.815			
	SEQ3	0.842			
	SEQ4	0.836			
INQ	INQ1	0.831	0.843	0.894	0.678
	INQ2	0.834			
	INQ3	0.818			
	INQ4	0.811			
SYQ	SYQ1	0.906	0.789	0.907	0.709
	SYQ2	0.822			
	SYQ3	0.826			
	SYQ4	0.812			

In this study, two criteria were used to examine discriminant validity. Firstly, the criterion developed by Fornell and Larcker (1981) was used. According to the findings, each construct’s AVE square root should be higher than its correlation with any other construct in the model. Table 4 reveals this criterion was met, hence discriminant validity exists.

**Table 4:** Fornell and Larcker’s test.

	**CI**	**CON**	**INQ**	**INT**	**PU**	**SAT**	**SEQ**	**SYQ**
CI	*0.854							
CON	**0.748	0.865						
INQ	0.757	0.709	0.823					
INT	0.714	0.751	0.77	0.844				
PU	0.736	0.844	0.652	0.682	0.853			
SAT	0.830	0.822	0.751	0.772	0.79	0.845		
SEQ	0.767	0.68	0.811	0.763	0.633	0.734	0.825	
SYQ	0.749	0.709	0.815	0.738	0.658	0.746	0.812	0.842

*Numbers on the leading diagonal are the square root of AVE for each construct, **correlation among the constructs. PU, perceived usefulness; CON, confirmation; INT, interaction; SAT, Satisfaction; CI, Continuance Intention; INQ, Information Quality; SYQ: System Quality, SEQ: Service Quality.

Secondly, this study conducted the heterotrait–monotrait ratio (HTMT) approach to test discriminant validity (Henseler et al., 2015). As shown in Table 5, all values are ≤0.85. The existence of discriminant validity and the findings of the Fornell-Larcker criterion are therefore corroborated. In conclusion, the validity and reliability of each instrument employed in this investigation were found to be adequate.

**Table 5:** HTMT.

	CI	CON	INQ	INT	PU	SAT	SEQ	SYQ
CI								
CON	0.799							
INQ	0.801	0.759						
INT	0.745	0.798	0.811					
PU	0.786	0.812	0.697	0.722				
SAT	0.805	0.822	0.797	0.808	0.847			
SEQ	0.811	0.729	0.823	0.803	0.677	0.779		
SYQ	0.795	0.762	0.803	0.779	0.706	0.795	0.764	

PU, perceived usefulness; CON, confirmation; INT, interaction; SAT, satisfaction; CI, continuance Intention; INQ, information quality; SYQ, system quality; SEQ, service quality.

### 5.2. Inner model

Predictive relevance (*Q*^2^), explanatory power (*R*^2^), and proposed hypothesis were all evaluated using structural model analysis (Hair et al., 2019). Prior to analyzing the proposed structural relations, it is essential to examine collinearity to ensure that the regression results are unbiased. Accordingly, we assessed collinearity by calculating the variance inflation factor (VIF) values of the inner model, as suggested by Hair et al. (2019). It is recommended that VIF estimates be around or below 5 when checking for the lack of collinearity. According to Table 6, no collinearity issues were present because all constructs got a VIF of <5.

**Table 6:** Collinearity test.

	CI	CON	INQ	INT	PU	SAT	SEQ	SYQ
CI								
CON					1	4.531		
INQ						3.521		
INT						3.311		
PU	2.66			1		3.595		
SAT	2.66							
SEQ						4.612		
SYQ						4.44		

PU, perceived usefulness; CON, confirmation; INT, interaction; SAT, satisfaction; CI, continuance intention; INQ, information quality; SYQ, system quality; SEQ, service quality.

The proposed model’s predictive accuracy was measured using *R*^2^ and *Q*^2^. Following the advice of Hair et al. (2013), a 5,000 bootstrap re-samples technique was carried out to examine all proposed paths. Furthermore, estimates for *Q*^2^ were calculated using the blindfolding process. Table 7 shows that the proposed model has sufficient predictive accuracy because all dependent variables have *Q*^2^ estimates more than 0.

**Table 7:** Predictive accuracy.

Construct	*R* ^2^	*Q* ^2^
PU	0.713	0.572
INT	0.466	0.364
SAT	0.774	0.682
CI	0.740	0.631

PU, perceived usefulness; INT: interaction, SAT: satisfaction, CI: continuance intention, INQ: information quality, SYQ: system quality, SEQ: service quality.

According to Table 7, seven different constructs account for 74.0 percent of the CI variance (*R*^2^ = 0.740). Meanwhile, Six constructs were involved in the explanation of 74.4% of the SAT variance (*R*^2^ = 0.744). According to Chin (1998), such explanation power falls within the moderate to substantial category. To assess the predictive power, the PLS predict technique was also used (Hair et al., 2019). These outcomes, coupled with the fact that all of these variables had positive *Q*^2^ values, show the proposed model’s medium to high predictive accuracy.

Effect size (*f*^2^) measures if an independent latent variable has a substantial impact on a dependent latent variable. Values of 0.02, 0.15, 0.35 indicates the predictor variable’s low, medium, or large effect in the structural model (Cohen, 1988). This study focuses on the effect of exogenous latent variables on endogenous latent variables (CI and SAT). According to Table 8, the effect size of PU and SAT on the CI was 0.038 and 0.764, respectively, the effect size of CON, INQ, INT, PU, SEQ, SYQ on the SAT was 0.08, 0.2, 0.046, 0.076, 0.1, 0.1. Therefore, Therefore, the effect size of PU on CI is between low and medium, the effect size of SAT on CI is large. In addition, the effect size of CON, INT, PU, SEQ, SYQ on SAT is between low and medium, the effect size of INQ on SAT is between medium and large.

**Table 8:** Effect size (*f*^2^).

	CI	SAT
CON		0.08
INQ		0.2
INT		0.046
PU	0.038	0.076
SAT	0.764	
SEQ		0.1
SYQ		0.1

Based on the results of the path analysis presented in Figure 1 and Table 9, we can infer that H1–H5 and H7–H10 are supported, but hypothesis 6 is not. Compared with PU (*β* = 0.162, *p* = 0.000), SAT has a greater impact on CI. Compared to other constructs (PU: *β* = 0.248, *p* = 0.000; SYQ: *β* = 0.108, *p* = 0.000; INT: *β* = 0.186, *p* = 0.000; SEQ: *β* = 0.103, *p* = 0.000), CON (*β* = 0.286, *p* = 0.000) is the most important predictor of SAT. In addition, PU was positively correlated with INT (*β* = 0.682, *p* = 0.000). CON was positively correlated with PU (*β* = 0.844, *p* = 0.000).

**Table 9:** Path analysis results.

	Path	*β*	SD	*T* Statistics	Value of *p*	Result
H1	PU → CI	0.162	0.023	6.924	0.000	Supported
H2	PU → SAT	0.248	0.025	9.857	0.000	Supported
H3	PU → INT	0.682	0.014	47.819	0.000	Supported
H4	CON → PU	0.844	0.008	109.35	0.000	Supported
H5	CON → SAT	0.286	0.031	9.177	0.000	Supported
H6	INQ → SAT	0.056	0.037	1.515	0.13	Unsupported
H7	SYQ → SAT	0.108	0.03	3.579	0.000	Supported
H8	INT → SAT	0.186	0.028	6.719	0.000	Supported
H9	SEQ → SAT	0.103	0.029	3.613	0.000	Supported
H10	SAT → CI	0.727	0.023	31.785	0.000	Supported

PU, perceived usefulness; CON, confirmation; INT, interaction; SAT, satisfaction; CI, continuance intention; INQ, information quality; SYQ, system quality; SEQ, service quality; SD, standard deviation.

### 5.3. Multi-group analysis

According to Matthews (2017), before performing a multi-group analysis, it is necessary to determine that the sample size of each subgroup must be large enough to meet statistical power guidelines. In this study, the sample sizes were 537. Following the more rigorous recommendations from a power analysis, 75 observations per group are needed to detect *R*^2^ values of around 0.25 at a significance level of 5% and a power level of 80%. Therefore, the group-specific sample sizes can be considered sufficiently large (Cohen, 1992).

The next procedure is to conduct a test of measurement invariance. In PLS-SEM, the measurement invariance of composite models (MICOM) process is used to check for measurement invariance (Henseler et al., 2016). During this procedure, three steps should be included: configural invariance, compositional invariance, and equality of composite mean values and variances (Henseler et al., 2016). Firstly, configural invariance must be conducted in MICOM (Henseler et al., 2016). Its involved three criteria: (a) identical indicators per measurement model, (b) identical data treatment, and (c) identical algorithm settings or optimization criteria (Henseler et al., 2016). Since all of the aforementioned requirements have been satisfied, configural invariance has been proven.

Step two of MICOM is to investigate at compositional invariance, which occurs when composite scores are established uniformly across groups (Dijkstra and Henseler, 2011). According to Table 10, compositional invariance is obtained when the original correlations are equal to or higher than the 5.00% quantile correlation (given in the 5.00% column).

**Table 10:** MICOM Step 2 results report.

	Original correlation	Correlation permutation mean	5.00%	Permutation *p*-Values
PU	1	0.999	0.997	0.392
SAT	0.999	0.997	0.992	0.832

PU, perceived usefulness; SAT, satisfaction.

In the third phase of MICOM, composite equality of mean and variance in different groups should be checked. The mean original difference and variance original difference both need to be within the 95% confidence interval respectively, as illustrated in Table 11. All constructs that passed the measurement invariance test are further supported by the Permutation *p*-values (*M*) and Permutation *p*-values (*V*) >0.05 shown in Table 11.

**Table 11:** MICOM Step 3 results report.

	Mean-Original difference (male–female)	Mean-Permutation mean difference (male–female)	2.50%	97.50%	Permutation *p*-values (*M*)	Variance-original difference (male–female)	Variance-permutation mean difference (male–female)	2.50%	97.50%	Permutation *p*-values (*V*)
PU	−0.132	0	−0.086	0.088	0.221	0.303	−0.003	−0.127	0.422	0.089
SAT	−0.138	0	−0.087	0.083	0.311	0.29	−0.003	−0.136	0.328	0.077

PU, perceived usefulness; SAT, satisfaction.

Once the invariance is confirmed, the next step is to check if there are appreciable differences between the path coefficients of the theoretical models for the two sets of data. According to the Table 12, there is no gender gap in any of the relationships. Furthermore, the permutation *p*-values (>0.05) support this conclusion.

**Table 12:** Permutation test path coefficient results.

	Path coefficients original (male)	Path coefficients original (female)	Path coefficients original difference (male–female)	Path coefficients permutation mean difference (male–female)	2.50%	97.50%	Permutation *p*-values
PU → CI	0.186	0.147	0.039	−0.002	−0.093	0.095	0.429
SAT → CI	0.727	0.726	0.001	0.002	−0.093	0.092	0.981

PU, perceived usefulness; SAT, satisfaction; CI, continuance intention.

## 6. Discussion

The purpose of this study is to investigate the key predictors of online learning system CI. In the literature review, we introduce expectation-confirmation theory and the ISSM as the theoretical framework, and INT as the important predictor of the CI of online learning system. In addition, we hypothesized CI of online learning system is predicted by several constructs: PU, INT, CON, SAT, INQ, SYQ, SEQ. The model was validated by partial least square structural equation model technique. All hypotheses were supported except for the relationship between INT and SAT, accounting for 74.0% of the total variance in CI of online learning systems. After multi-group analysis, it was found that there was no significant gender difference between PU, SAT and CI of online learning system. In the following paragraphs, we will discuss the important findings of this study. In addition, Hypotheses H1, H2, and H3 gained empirical support. This confirms that PU is a determinant of CI, SAT and INT. That is to say, when students believe that online learning systems can effectively improve academic performance, their SAT will be increased, their CI will be increased. In addition, in online learning environments, valuable knowledge and ideas often stimulate the INT of student–student, student-teacher and student-content. These results confirm the findings of Wang et al. (2021), who found that PU is critical to the success of information systems (such as SAT, intention to continue use, etc.). In this study, to improve the CI, SAT and INT of college students’ online learning system, on the one hand, the learning content should be changed, so that students can get valuable information, so as to improve their academic performance and the ability to use knowledge to solve problems. On the other hand, improve the efficiency of online learning system, so that students can easily operate, save time.

Hypotheses H4 and H5 were accepted. The results showed that CON significantly influences PU and SAT. The findings demonstrated that PU and SAT are highly influenced by CON. Such results are consistent with earlier ECM-based investigations (Yang and Jiang, 2020). Additionally, it was discovered that CON was a stronger indicator of students’ SAT than PU (Niu and Wu, 2022a). This shows that satisfying students’ expectations for how online learning activities are performed is significantly more crucial to their SAT and may indirectly affect their intention to continue to use. In this study, in order to improve the SAT of college students’ online learning system, on the one hand, students’ demands for the online learning system should be extensively collected to meet their expectations. On the other hand, the functions of online learning system should be publicized to college students so that they can have a correct and comprehensive understanding of the learning system and form reasonable expectations.

The hypotheses H7 and H9 were supported by data. That is to say, the level of SAT with an online learning system is strongly related to the SYQ and the SEQ provided by the system. This demonstrates that people who utilize online learning systems are more content with their jobs and more productive when they have a positive perception of their utilization. It is predicted that the higher the quality of the used online learning system, the more satisfied the system’s end users will be with it. The results of this study support and extend Ajzen (1991) Theory of Reasoned Action (TRA), according to which a person uses an information system if using it would result in benefits for himself. Additionally, user SAT increases in direct proportion to the quality of services offered by the online learning system (Sasono and Novitasari, 2020). The research undertaken by Wang (2008) to examine the success of e-commerce in Taiwan and Wang and Liao (2008) to examine the success of e-government in Taiwan is confirmed and expanded by the findings of this study. Between SEQ and customer SAT, both studies demonstrate a significant and positive association.

INQ is not a very important predictor of student SAT in an online learning context, as evidenced by the lack of support for hypothesis H6. The findings of Eom et al. (2012) are at odds with this outcome, which found that user SAT was significantly correlated with the quality of the information provided to them based on the D&M information system success model for e-learning. The reason for the inconsistent results may be that, in the context of COVID-19, students passively accept online learning, while the frequency and quality of INT (student–student INT, student-teacher INT, and student-content INT) decrease, leading to the shift of factors affecting students’ SAT to hardware (computer, network, etc.) and learning system characteristics (SEQ, SYQ, PU).

H8 was supported by the data. That is to say, INT (student–student INT, student-teacher INT, student-content INT) is an important predictor of online learning SAT. This result is consistent with Ares Albirru (2021), which detailed the interactive learning context and discovered that the INT has an impact on the degree of students’ SAT. Based on the results of this research, the level of user SAT with an online learning system increases as both the quality and frequency of INTs increase inside that system. In this study, one of the most important ways to improve college students’ online learning SAT is to strengthen INT. On the one hand, student–student INT can realize knowledge sharing, information collision, and generate the meaning construction of knowledge; On the other hand, instructor-student INT can realize knowledge transmission, student feedback, and change the way of knowledge transmission.

Since H10 was supported, and students’ SAT is a crucial indicator of the CI. That is to say, the higher the students’ SAT with the online learning system, the stronger their CI, and the more likely they are to have continuous use behavior. The findings of Cheng (2019) are confirmed by these findings, which indicated that students’ SAT is a significant predictor of their CI based on ECM and TTF. In this study, the more satisfied users are with an online learning system, the more likely they are to continue using it. In this study, to improve college students’ SAT with online learning system, they can start from the following aspects, such as learning materials, learning system and teacher support, so as to enhance their CI.

In addition, there was no significant gender difference in the relationship between PU and CI and in the relationship between SAT and CI. The above results are consistent with Tam (2020), which studied the intention of continuous use of mobile banking in Vietnam based on the TAM, the TTF and the ECM, and concluded that there was no gender difference in the relationship between SAT and CI, and there was no gender difference in the impact of PU on the CI (Wu and Wang, 2020). In other words, gender does not need to be considered in the process of enhancing students’ CI in online learning systems usage.

## 7. Implications

In the context of online learning, this study creates a research model that explains 77.4% and 74.0% of the variance in users’ SAT and CI using the system, respectively. As a result, the research model for this study has greater predictive power in relationship to the gathered information. The next section includes specific theoretical and practical implications.

### 7.1. Theoretical implications

Firstly, the proposed model makes an effort to establish an unified framework based on the three fundamental viewpoints (namely, ECM, ISSM, and INT) that may affect the CI of online learning. The findings demonstrated conclusively that the research model adequately supported the primary aim of the study, namely, that the integrated model provides a sufficient theoretical framework to explain online learners’ CI of the online learning system. Secondly, the findings of this study demonstrate the importance of ECM in determining students’ SAT with their intention to continue using the online learning system. In the meantime, the findings indicate that students place a disproportionate amount of emphasis on their CON of expectations toward the system when establishing their SAT with the system, which is the most important direct driver of their intention to continue using the system, and that the salience of SAT is significantly greater than PU. The results also suggest that user SAT is the most important factor in determining the intention to continue using an information system, as users lay a greater focus on the CON of their expectations than on their post-adoption beliefs when determining their levels of SAT. As a result, it is essential to consider students’ level of contentment while analyzing their propensity to engage in further online education. This result recommends that in order to increase students’ intention to continue using the online learning system, academic institutions should first focus on identifying sources of students’ disconfirmation and then rethink how to strive to optimize their SAT by reassuring their expectations and supporting their efficient system use.

### 7.2. Practical implications

This study will benefit online learning systems by increasing student engagement and efficacy. Firstly, an online learning system should meet the needs of its users by giving them useful and interesting information and encouraging INT between learners. To better meet students’ demands, online learning systems should understand their users. Additionally, the intention of learners to continue using and advocating online learning is also strongly influenced by their level of SAT. Secondly, CON also accurately predicts how satisfied students are with online education. Because of this, it is necessary to educate students on how to utilize the online learning system effectively in order to enhance their CON of expectations and further maximize their SAT. Finally, Online instructors should employ vivid examples, appealing instructional techniques, and valuable and engaging content to encourage participant engagement. With the help of these methods, teachers may be able to meet their students’ needs and expectations.

## 8. Conclusion

The purpose of this study is to investigate the key predictors of online learning system CI. The results show that CON, PU, INT, SYQ, and SEQ are effective predictors of the CI of online learning system, while there is no significant positive correlation between the INQ and the SAT of an online learning system. In addition, there were no significant gender differences in PU, SAT and CI of online learning system. This study extends the field of CI of online learning system. On the one hand, the proposed model makes an effort to establish an unified framework based on the three fundamental viewpoints (namely, ECM, ISSM, and INT) that may affect the CI of online learning. On the other hand, the findings of this study demonstrate the importance of ECM in determining students’ SAT with their intention to continue using the online learning system. Additionally, this study also provide some suggestions for instructors of online learning and decision-makers.

On the basis of the current study’s limitations, potential directions for further research are recommended. The first recommendation concerns the design of the survey. Without a doubt, the process should be set up to protect personal information and keep people from having to fill out the same questionnaires over and over again. Future studies using qualitative interviews or case studies may be able to confirm these empirical findings, as the survey results do not allow for the inference of causality. Secondly, Our sample consists of learners who engaged in online education throughout the COVID-19 epidemic. This indicates that these students’ perspective toward online learning is wholly passive. When learning is transferred from passive students to active students, entirely new outcomes may be attained. The variables affecting students’ SAT and intent to continue learning online would be entirely different for those who actively engage in this mode of instruction. As a comparison to the existing sample, their perspectives are worthwhile researching. Additionally, attitudes among students in various disciplines and courses may vary. Thirdly, this study only explores that there is no significant gender difference in the relationship between college students’ PU, SAT, and CI of online learning system. But why there is no significant gender difference in these two relationships has not been explored. Future studies can be designed to continue to specifically examine the role of gender in these relationships. Fourthly, In view of the impact of the novel coronavirus epidemic, convenience sampling method was adopted in this study, which on the one hand may lead to higher sampling error, and on the other hand may cause the results of the study cannot be generalized to a larger research field. In the future, the COVID-19 epidemic will be over. It is suggested that researchers adopt random sampling or other more scientific sampling methods to obtain results with lower sampling error and more generalization validity. Finally, The effectiveness of students’ learning was not examined in this study, despite the fact that our qualitative findings suggest that it is a major concern. As a result, we anticipate further study on measuring students’ learning as a result of extensive online instruction. The creation of a wide range of reliable evaluation tools is another area deserving of study.

## Data availability statement

The raw data supporting the conclusions of this article will be made available by the authors, without undue reservation.

## Author contributions

JZ and LZ: conceptualization and writing original draft. MZ and YL: data curation. JZ and MZ: writing–review and editing. All authors contributed to the article and approved the submitted version.

## Funding

This study is funded by Key Project of Ministry of Education of China in 2018, No: DCA180322.

## Conflict of interest

The authors declare that the research was conducted in the absence of any commercial or financial relationships that could be construed as a potential conflict of interest.

## Publisher’s note

All claims expressed in this article are solely those of the authors and do not necessarily represent those of their affiliated organizations, or those of the publisher, the editors and the reviewers. Any product that may be evaluated in this article, or claim that may be made by its manufacturer, is not guaranteed or endorsed by the publisher.

## Appendix A

**Table:**

**Construct**		**Items**	**Source**
Perceived usefulness		Using online learning enables me to accomplish my tasks more quickly	Mohammadi (2015)
		Using online learning improves my learning performance	
		Using online learning helps me learn effectively	
		Overall online learning is useful	
Confirmation		My experience with using online learning was better than what I expected	Bhattacherjee (2001)
		The service level provided by online learning was better than what I expected	
		Overall, most of my expectations from using online learning were confirmed	
Interaction	INSTRUCTORS-STUDENTS	The instructor is supportive when a student had difficulties or questions	Chung and Chen (2020)
		The instructor provided clear, constructive feedback for student learning	
	STUDENTS-STUDENTS	The course fosters student-to-student interaction for supporting learning	
		The course utilizes effective strategies that engage students in learning	
	CONTENTS-STUDENTS	The course content provides mutual interaction to facilitate student learning	
		The course facilitates student achievement of the stated course objectives	
Satisfaction		My decision to use the online learning was a wise one	Isaac et al. (2019)
		The online learning has met my expectations	
		Overall, I am satisfied with the online learning	
Continuance intention		I intend to continue using the cloud-based e-learning system in the future	Cheng (2019)
		I will use the cloud-based e-learning system on a regular basis in the future	
		I will frequently use the cloud-based e-learning system in the future	
		My intentions are to continue using the cloud-based e-learning system than use any alternative means	
Service quality		The responsible service personnel are always highly willing to help whenever I need support with the e-learning system	Urbach (2010)
		The responsible service personnel provide personal attention when I experience problems with the e-learning system	
		The responsible service personnel provide services related to the e-learning system at the promised time	
		The responsible service personnel have sufficient knowledge to answer my questions in respect to the e-learning system	
Information quality		The information provided by e-learning system is useful	
		The information provided by e-learning system is understandable	
		The information provided by e-learning system is interesting	
		The information provided by e-learning system is reliable	
System quality		The e-learning system is easy to navigate	
		The e-learning system allows me to easily find the information I am looking for	
		The e-learning system is well structured	
		The e-learning system is easy to use	

## References

[ref1] Abou-Khalil V. Helou S. Khalifé E. Chen M. A. Majumdar R. Ogata H. (2021). Emergency online learning in low-resource settings: effective student engagement. Strategies 11:24. doi: 10.3390/educsci11010024

[ref2] Ahmad A. Rasul T. Yousaf A. Zaman U. (2020). Understanding factors influencing elderly diabetic patients’ continuance intention to use digital health wearables: extending the technology acceptance model (TAM). J. Open Innovation: Technol. Market Complexity 6:81 Available at: https://www.mdpi.com/2199-8531/6/3/81

[ref3] Ahn T. Ryu S. Han I. (2004). The impact of the online and offline features on the user acceptance of internet shopping malls. Electron. Commer. Res. Appl. 3, 405–420. doi: 10.1016/j.elerap.2004.05.001

[ref4] Ajzen I. (1991). The theory of planned behavior. Organ. Behav. Hum. Decis. Process. 50, 179–211. doi: 10.1016/0749-5978(91)90020-T

[ref5] Al-Adwan A. S. Albelbisi N. A. Hujran O. Al-Rahmi W. M. Alkhalifah A. (2021). Developing a holistic success model for sustainable E-learning: a structural equation modeling approach. Sustainability 13:9453. doi: 10.3390/su13169453

[ref6] Al-Adwan A. S. Nofal M. Akram H. Albelbisi N. A. Al-Okaily M. (2022a). Towards a sustainable adoption of E-learning systems: the role of self-directed. Learning 21, 245–267. doi: 10.28945/4980

[ref7] Al-Adwan A. S. Yaseen H. Alsoud A. Abousweilem F. Al-Rahmi W. M. (2022b). Novel extension of the UTAUT model to understand continued usage intention of learning management systems: the role of learning tradition. Educ. Inf. Technol. 27, 3567–3593. doi: 10.1007/s10639-021-10758-y

[ref8] Alismaiel O. A. Cifuentes-Faura J. Al-Rahmi W. M. (2022). Online learning, Mobile learning, and social media technologies: an empirical study on constructivism theory during the COVID-19 pandemic. Sustainability 14:11134. Available at: https://www.mdpi.com/2071-1050/14/18/11134

[ref9] Alksasbeh M. Abuhelaleh M. Almaiah M. AL-jaafreh M. Abu Karaka A. (2019). “Towards a model of quality features for mobile social networks apps in learning environments: An extended information system success model,” in International Association of Online Engineering. Retrieved February 3, 2023 from https://www.learntechlib.org/p/209780/

[ref10] Alyoussef I. (2021). E-learning system use during emergency: An empirical study during the COVID19 pandemic. Front. Educ. 6:677753. doi: 10.3389/feduc.2021.677753

[ref11] Anderson J. C. Gerbing D. W. (1988). Structural equation modeling in practice: a review and recommended two-step approach. Psychol. Bull. 103, 411–423. doi: 10.1037/0033-2909.103.3.411

[ref12] Ares Albirru E. A., (2021). “Perceived satisfaction and perceived usefulness of E-learning: the role of interactive learning and social influence,” in *Proceedings of the 3rd International Conference on Educational Development and Quality Assurance (ICED-QA 2020)*.

[ref13] Ashrafi A. Zareravasan A. Rabiee Savoji S. Amani M. (2020). Exploring factors influencing students’ continuance intention to use the learning management system (LMS): a multi-perspective framework. Interact. Learn. Environ. 30, 1475–1497. doi: 10.1080/10494820.2020.1734028

[ref14] Baber H. (2020). Determinants of students’ perceived learning outcome and satisfaction in online learning during the pandemic of COVID19. J. Educ. e-Learn. Res. 7, 285–292. doi: 10.20448/journal.509.2020.73.285.292

[ref15] Baranova T. Kobicheva A. Tokareva E. (2022). Factors influencing students’ continuance intention to learn in blended environments at university. Electronics 11:2069. Available at: https://www.mdpi.com/2079-9292/11/13/2069

[ref16] Bhattacherjee A. (2001). Understanding information systems continuance: an expectation-confirmation model. MIS Q. 25, 351–370. doi: 10.2307/3250921

[ref17] Chang I. H. Chen R.-S. (2020). The impact of perceived usefulness on satisfaction with online parenting resources: the mediating effects of liking and online interaction. Asia Pac. Educ. Res. 29, 307–317. doi: 10.1007/s40299-019-00484-y

[ref18] Chen T. Peng L. Yin X. Rong J. Yang J. Cong G. (2020). Analysis of user satisfaction with online education platforms in China during the COVID-19 pandemic. Healthcare (Basel) 8:200. doi: 10.3390/healthcare8030200, PMID: 32645911 PMC7551570

[ref19] Cheng Y.-M. (2019). How does task-technology fit influence cloud-based e-learning continuance and impact? Educ. + Training 61, 480–499. doi: 10.1108/et-09-2018-0203

[ref01] Cheng M. Yuen A. H. K. (2018). Student continuance of learning management system use: A longitudinal exploration. Comput. Educ. 120, 241–253. doi: 10.1016/j.compedu.2018.02.004

[ref09] Cheng X. Yang S. Zhou S. (2019). Why do college students continue to use mobile learning? Learning involvement and self-determination theory. Br. J. Educ. Technol. 50, 626–637. doi: 10.1111/bjet.12634

[ref20] Chin W. W. (1998). The partial least squares approach to structural equation modeling. Modern Methods Bus. Res. 295, 295–336.

[ref21] Chung J. Chen H.-C. (2020). Development and psychometric properties of student perceptions of an online course (SPOC) in an RN-to-BSN program. Nurse Educ. Today 85:104303. doi: 10.1016/j.nedt.2019.104303, PMID: 31785574

[ref22] Cifuentes-Faura J. Obor D. To L. Al-Naabi I. (2021). Cross-cultural impacts of COVID-19 on higher education learning and teaching practices in Spain, Oman, Nigeria and Cambodia: a cross-cultural study. J. Univ. Teach. Learn. Pract. 18, 1–18. doi: 10.53761/1.18.5.8

[ref23] Cohen J. (1988). Statistical power analysis for the behavioral sciences. 2nd Edn. New York: Routledge.

[ref24] Cohen J. A. (1992). A power primer. Psychol. Bull. 112, 155–159. doi: 10.1037/0033-2909.112.1.155, PMID: 19565683

[ref25] Croxton R. A. (2014). The role of interactivity in student satisfaction and persistence in online learning. J. Online Learn. Teach. 10, 314–325.

[ref26] Dai H. M. Teo T. Rappa N. A. Huang F. (2020). Explaining Chinese university students’ continuance learning intention in the MOOC setting: a modified expectation confirmation model perspective. Comput. Educ. 150:103850. doi: 10.1016/j.compedu.2020.103850

[ref27] Dalvi-Esfahani M. Wai Leong L. Ibrahim O. Nilashi M. (2020). Explaining students’ continuance intention to use Mobile web 2.0 learning and their perceived learning: an Integrated approach. J. Educ. Comput. Res. 57, 1956–2005. doi: 10.1177/0735633118805211

[ref28] Davis F. D. (1989). Perceived usefulness, perceived ease of use, and user acceptance of information technology. MIS Q. 13, 319–340. doi: 10.2307/249008

[ref29] DeLone W. H. McLean E. R. (1992). Information systems success: the quest for the dependent variable. J. Info. Syst. Res. 3, 60–95. doi: 10.1287/isre.3.1.60

[ref30] DeLone W. H. McLean E. R. (2002). “Information systems success revisited,” in *Proceedings of the 35th annual Hawaii international conference on system sciences*.

[ref31] Dijkstra T. K. Henseler J. (2011). Linear indices in nonlinear structural equation models: best fitting proper indices and other composites. Qual. Quant. 45, 1505–1518. doi: 10.1007/s11135-010-9359-z

[ref32] Efiloğlu Kurt Ö. (2019). Examining an e-learning system through the lens of the information systems success model: empirical evidence from Italy. Educ. Inf. Technol. 24, 1173–1184. doi: 10.1007/s10639-018-9821-4

[ref33] Eom S. Ashill N. J. Arbaugh J. Stapleton J. L. (2012). The role of information technology in e-learning systems success. Hum. Syst. Manag. 31, 147–163. doi: 10.3233/HSM-2012-0767

[ref34] Faura-Martínez U. Lafuente-Lechuga M. Cifuentes-Faura J. (2022). Sustainability of the Spanish university system during the pandemic caused by COVID-19. Educ. Rev. 74, 645–663. doi: 10.1080/00131911.2021.1978399

[ref35] Fornell C. Larcker D. F. (1981). Evaluating structural equation models with unobservable variables and measurement error. J. Mark. Res. 18, 39–50. doi: 10.1177/002224378101800104

[ref06] Fu X. Yan T. Tian Y. Niu X. Xu X. Wei Y.. (2022). Exploring factors influencing students’ entrepreneurial intention in vocational colleges based on structural equation modeling: evidence from china. Front. Psychol. 13:898319. doi: 10.3389/fpsyg.2022.898319 35747685 PMC9211024

[ref36] Garrison D. R. Anderson T. Archer W. (2003). A theory of critical inquiry in online distance education. Handbook Distance Educ. 1, 113–127.

[ref015] Gefen D. Karahanna E. Straub D. W. (2003). Trust and TAM in online shopping: an integrated model. MIS Q. 27, 51–90. doi: 10.2307/30036519

[ref37] Gravetter F. J. Forzano L.-A. B. (2018). Research methods for the behavioral sciences. Boston: Cengage learning.

[ref38] Gupta A. Dhiman N. Yousaf A. Arora N. (2021). Social comparison and continuance intention of smart fitness wearables: an extended expectation confirmation theory perspective. Behav. Inform. Technol. 40, 1341–1354. doi: 10.1080/0144929X.2020.1748715

[ref011] Hair J. F. Hult G. T. M. Ringle C. M. Sarstedt M. Danks N. P. Ray S. (2021). “An introduction to structural equation modeling,” in Partial Least Squares Structural Equation Modeling (PLS-SEM) Using R. Springer, Cham: Classroom Companion: Business.

[ref39] Hair J. F. Ringle C. M. Sarstedt M. (2013). Partial least squares structural equation modeling: rigorous applications, better results and higher acceptance. Int. J. Strategic Manage. 46, 1–12. doi: 10.1016/j.lrp.2013.01.001

[ref40] Hair J. F. Risher J. J. Sarstedt M. Ringle C. M. (2019). When to use and how to report the results of PLS-SEM. Eur. Bus. Rev. 31, 2–24. doi: 10.1108/EBR-11-2018-0203

[ref41] Halilovic S. Cicic M. (2013). Antecedents of information systems user behaviour – extended expectation-confirmation model. Behav. Inform. Technol. 32, 359–370. doi: 10.1080/0144929X.2011.554575

[ref42] Henseler J. Ringle C. M. Sarstedt M. (2015). A new criterion for assessing discriminant validity in variance-based structural equation modeling. J. Acad. Mark. Sci. 43, 115–135. doi: 10.1007/s11747-014-0403-8

[ref43] Henseler J. Ringle C. M. Sarstedt M. (2016). Testing measurement invariance of composites using partial least squares. Int. Mark. Rev. 33, 405–431. doi: 10.1108/IMR-09-2014-0304

[ref44] Hsu L. (2021). What makes good LMOOCs for EFL learners? Learners’ personal characteristics and information system success model. Comput. Assist. Lang. Learn. 1-25, 1–25. doi: 10.1080/09588221.2021.1899243

[ref45] Iqbal M. Rafiq M. Soroya S. H. (2022). Examining predictors of digital library use: an application of the information system success model. Electron. Libr. 40, 359–375. doi: 10.1108/EL-01-2022-0008

[ref46] Isaac O. Aldholay A. Abdullah Z. Ramayah T. (2019). Online learning usage within Yemeni higher education: the role of compatibility and task-technology fit as mediating variables in the IS success model. Comput. Educ. 136, 113–129. doi: 10.1016/j.compedu.2019.02.012

[ref47] Jiang L. Li K. (2020). “Research on the influencing factors of music virtual community based on expectation confirmation theory,” in *2020 IEEE international conference on information technology, big data and artificial intelligence (ICIBA)*.

[ref48] Jin Y. Q. Lin C. L. Zhao Q. Yu S. W. Su Y. S. (2021). A study on traditional teaching method transferring to E-learning under the Covid-19 pandemic: from Chinese Students' perspectives. Front. Psychol. 12:632787. doi: 10.3389/fpsyg.2021.632787, PMID: 33776854 PMC7991594

[ref49] Kerman N. T. Banihashem S. K. Noroozi O. Biemans H. J. (2022). “The effects of students perceived usefulness and trustworthiness of peer feedback on learning satisfaction in online learning environments,” in *8th international conference on higher education advances, HEAd 2022*.

[ref50] Klobas J. E. McGill T. J. (2010). The role of involvement in learning management system success. J. Comput. High. Educ. 22, 114–134. doi: 10.1007/s12528-010-9032-5

[ref03] Kovačević I. Anđelković Labrović J. Petrović N. Kužet I. (2021). Recognizing predictors of students’ emergency remote online learning satisfaction during COVID-19. Educ. Sci. 11:693. doi: 10.3390/educsci11110693

[ref51] Lee M.-C. (2010). Explaining and predicting users’ continuance intention toward e-learning: an extension of the expectation–confirmation model. Comput. Educ. 54, 506–516. doi: 10.1016/j.compedu.2009.09.002

[ref52] Limayem M. Hirt S. G. Cheung C. M. (2007). How habit limits the predictive power of intention: the case of information systems continuance. MIS Q. 31:705. doi: 10.2307/25148817

[ref53] Liu N. Pu Q. (2020). Factors influencing learners’ continuance intention toward one-to-one online learning. Interact. Learn. Environ. 1-22, 1–22. doi: 10.1080/10494820.2020.1857785

[ref54] Lu Y. Wang B. Yaobin L. (2019). Understanding key drivers of MOOC satisfaction and continuance intention to use. J. Electron. Commer. Res. 20:13.

[ref55] Lu J. Yang J. Yu C.-S. (2013). Is social capital effective for online learning? Inf. Manag. 50, 507–522. doi: 10.1016/j.im.2013.07.009

[ref56] Majchrzak A. Beath C. M. Lim R. A. Chin W. W. (2005). Managing client dialogues during information systems design to facilitate client learning. MIS Q. 29:653. doi: 10.2307/25148704

[ref012] Matthews L. (2017). “Applying multigroup analysis in PLS-SEM: A step-by-step process” in Partial Least Squares Path Modeling. eds. Latan H. Noonan R. (Cham: Springer).

[ref57] McKinney V. Yoon K. Zahedi F. M. (2002). The measurement of web-customer satisfaction: an expectation and disconfirmation approach. Inf. Syst. Res. 13, 296–315. doi: 10.1287/isre.13.3.296.76

[ref58] Mohammadi H. (2015). Investigating users’ perspectives on e-learning: an integration of TAM and IS success model. Comput. Hum. Behav. 45, 359–374. doi: 10.1016/j.chb.2014.07.044

[ref59] Moore M. G. (1989). Editorial: three types of interaction. Am. J. Dist. Educ. 3, 1–7. doi: 10.1080/08923648909526659

[ref60] Moore M. G. Kearsley G. (1996). Distance education: a system view. Belmont, CA: Wadsworth.

[ref014] Niu X. Niu Z. Wang M. Wu X. (2022b). What are the key drivers to promote entrepreneurial intention of vocational college students? An empirical study based on structural equation modeling. Front. Psychol. 13:1021969. doi: 10.3389/fpsyg.2022.1021969 36389516 PMC9650398

[ref013] Niu X. Wu X. (2022a). Factors influencing vocational college students’ creativity in online learning during the COVID-19 pandemic: The group comparison between male and female. Front. Psychol. 13:967890. doi: 10.3389/fpsyg.2022.967890 36033061 PMC9404691

[ref61] Nuryanti Y. Hutagalung D. Nadeak M. Abadiyah S. Novitasari D. (2021). Understanding the links between system quality, information quality, service quality, and user satisfaction in the context of online learning. Int. J. Social Manage. Stud. 2, 54–64. doi: 10.5555/ijosmas.v2i4.51

[ref62] Oliver R. L. (1980). A cognitive model of the antecedents and consequences of satisfaction decisions. J. Mark. Res. 17, 460–469. doi: 10.1177/002224378001700405

[ref63] Oliver R. L. Bearden W. O. (1985). Disconfirmation processes and consumer evaluations in product usage. J. Bus. Res. 13, 235–246. doi: 10.1016/0148-2963(85)90029-3

[ref010] Petter S. Straub D. Rai A. (2007). Specifying formative constructs in information systems research. MIS Q. 31, 623–656. doi: 10.2307/25148814

[ref64] Pham L. Limbu Y. B. Bui T. K. Nguyen H. T. Pham H. T. (2019). Does e-learning service quality influence e-learning student satisfaction and loyalty? Evidence from Vietnam. Int. J. Educ. Technol. High. Educ. 16:7. doi: 10.1186/s41239-019-0136-3

[ref65] Ramadiani E. A. (2019). An integrated model of e-learning continuance intention in Indonesia. Int. J. Innovation Learn. 26, 1–26. doi: 10.1504/IJIL.2019.10021086

[ref66] Ringle C. M. Wende S. Becker J.-M. (2015). *SmartPLS 3*.

[ref67] Sasono I. Novitasari D. (2020). Enhancing service quality through innovation capabilities and work productivity. Int. J. Sci. Manage. Stud. 3, 123–133. doi: 10.51386/25815946/ijsms-v3i6p111

[ref68] Sher A. (2009). Assessing the relationship of student-instructor and student-student interaction to student learning and satisfaction in web-based online learning environment. J. Interact. Online Learn. 8, 102–120.

[ref69] Soria-Barreto K. Ruiz-Campo S. Al-Adwan A. S. Zuniga-Jara S. (2021). University students intention to continue using online learning tools and technologies: an international comparison. Sustainability 13:13813. doi: 10.3390/su132413813

[ref02] Supriyadi D. Safitri S. Kristiyanto D. (2020). Higher education e-Learning usability analysis using system usability scale. Int. J. Inf. Technol. 4, 436–446.

[ref70] Tam L. (2020). Factors affecting users’ continuance intention towards mobile banking in Vietnam. Am. J. Multidiscip. Res. Dev. 2, 42–51.

[ref71] Tan P. S. H. Choong Y. O. Chen I. C. (2021). The effect of service quality on behavioural intention: the mediating role of student satisfaction and switching barriers in private universities. J. Appl. Res. Higher Educ. 14, 1394–1413. doi: 10.1108/JARHE-03-2021-0122

[ref72] Teddy C. Martha M. N. (2018). The effect of service quality on student satisfaction and student loyalty: an empirical study. J. Social Stud. Educ. Res. 9, 109–131.

[ref04] Tsang J. T. Y. So M. K. P. Chong A. C. Y. Lam B. S. Y. Chu A. M. Y. (2021). Higher education during the pandemic: The predictive factors of learning effectiveness in COVID-19 Online Learning. Educ. Sci. 11:446. doi: 10.3390/educsci11080446

[ref73] Turnbull D. Chugh R. Luck J. (2021). Transitioning to E-learning during the COVID-19 pandemic: how have higher education institutions responded to the challenge? Educ. Inf. Technol. (Dordr) 26, 6401–6419. doi: 10.1007/s10639-021-10633-w, PMID: 34177349 PMC8220880

[ref74] Urbach (2010). An empirical investigation of employee portal success. J. Strateg. Inf. Syst. 19, 184–206. doi: 10.1016/j.jsis.2010.06.002

[ref05] Venkatesh V. Morris M. G. Davis G. B. Davis F. D. (2003). User acceptance of information technology: Toward a unified view. MIS Q. 27, 425–478. doi: 10.2307/30036540

[ref75] Wang Y. S. (2008). Assessing e-commerce systems success: a respecification and validation of the DeLone and McLean model of IS success. Inf. Syst. J. 18, 529–557. doi: 10.1111/j.1365-2575.2007.00268.x

[ref76] Wang Y. S. Liao Y. W. (2008). Assessing eGovernment systems success: a validation of the DeLone and McLean model of information systems success. Gov. Inf. Q. 25, 717–733. doi: 10.1016/j.giq.2007.06.002

[ref77] Wang T. Lin C.-L. Su Y.-S. (2021). Continuance intention of university students and online learning during the COVID-19 pandemic: a modified expectation confirmation model perspective. Sustainability 13:4586. doi: 10.3390/su13084586

[ref07] Widjaja A. Widjaja Y. G. (2022). The influence of interaction, learner characteristics, perceived usefulness, and perceived satisfaction on continuance intention in e-learning system. IJRBS. 2147-4478 11, 381–390. doi: 10.20525/ijrbs.v11i2.1665

[ref78] William H. D. Ephraim R. M. (2003). The DeLone and McLean model of information systems success: a ten-year update. J. Manag. Inf. Syst. 19, 9–30. doi: 10.1080/07421222.2003.11045748

[ref79] Wilson N. Keni K. Tan P. H. P. (2021). The role of perceived usefulness and perceived ease-of-use toward satisfaction and trust which influence computer consumers’ loyalty in China. Gadjah Mada Int. J. Bus. 23, 262–294. doi: 10.3316/informit.147511565887487

[ref08] Wu X. Wang M. (2020). Influence of professional identity and core self-evaluation on job satisfaction of vocational education teachers and the mediating effect of work stress. Rev. Argentina de Clin. Psicol. 29:31. doi: 10.24205/03276716.2020.204

[ref80] Xu F. Du J. T. (2021). Research on the drivers of undergraduates' intention to use university digital libraries: affinity theory as an additional construct of information system success model. Library Hi Tech. (ahead-of-print). doi: 10.1108/LHT-03-2021-0108

[ref81] Yan M. Filieri R. Gorton M. (2021). Continuance intention of online technologies: a systematic literature review. Int. J. Inf. Manag. 58:102315. doi: 10.1016/j.ijinfomgt.2021.102315

[ref82] Yang D.-S. Jiang K. (2020). Research on the influencing factors of the continuance intention online education platforms: based on expectation confirmation theory. J. Manage. Humanity Res. 3, 61–70. doi: 10.22457/jmhr.v03a06106

[ref83] Yildiz Durak H. (2018). Flipped learning readiness in teaching programming in middle schools: modelling its relation to various variables. J. Comput. Assisted Learn. 34, 939–959. doi: 10.1111/jcal.12302

[ref84] Zeithaml V. A. (1988). Consumer perceptions of price, quality, and value: a means-end model and synthesis of evidence. J. Mark. 52, 2–22. doi: 10.1177/002224298805200302

